# Aspirin as Primary Prevention of Acute Coronary Heart Disease Events

**DOI:** 10.9734/BJMMR/2014/11212

**Published:** 2014-07-25

**Authors:** Stephen P. Glasser, Martha Hovater, Todd M. Brown, George Howard, Monika M. Safford

**Affiliations:** 1 Division of Preventive Medicine University of Alabama at Birmingham, 171711th Avenue South, Birmingham, AL, USA.; 2 Department of Biostatistics, University of Alabama at Birmingham, USA.

**Keywords:** Aspirin, prophylaxis, incident, coronary, heart, disease

## Abstract

**Background/Objective:**

Aspirin for primary prophylaxis is controversial. This study evaluated associations between prophylactic aspirin use and incident acute coronary heart disease (CHD) events.

**Methods and Results:**

The Reasons for Geographic and Racial Differences in Stroke (REGARDS) Study was accessed for aspirin use examining black and white hazards for incident CHD, for men and women, each adjusting incrementally for sampling, sociodemographics, and CHD risk factors. Stratified models examined risks across strata of the Framingham risk score, and all-cause mortality. 23,949 participants (mean 64 yo), had 503 incident events over a 3.5 year follow-up. Prophylactic aspirin use was not associated with incident acute CHD, HR 1.05 (95% CI 0.86, 1.29). Modeling had little impact on the HR (1.09 {95% CI 0.89, 1.33) nor did the addition of risk factors (HR 1.00 {95% CI 0.81, 1.23). Aspirin use was not associated with incident CHD for any Framingham risk level. Findings were similar when including all aspirin users (not just those taking aspirin prophylactically), and when examining associations with all-cause mortality. There was no excess hospitalized bleeding in the aspirin users.

**Conclusion:**

Aspirin was not associated with lower risk for incident acute CHD overall, or within race, gender, or Framingham Risk Score.

## 1. INTRODUCTION

Aspirin is an effective anti-platelet and anti-inflammatory agent [1]. In a meta-analysis of trials of aspirin in the secondary prevention of cardiovascular and cerebrovascular events, aspirin significantly reduced the number of strokes and myocardial infarctions (MI) [2]. The data for long-term primary prevention are less clear. Nevertheless, aspirin is currently recommended as primary prevention for some men and women, particularly those over age 65 [3]. The use of aspirin for primary prevention is still undergoing study in at least 1 ongoing intervention trial [4]. It has been suggested that lower dose aspirin might mitigate some of the bleeding complications attendant with aspirin use, but, the effectiveness of a lower dose remains uncertain, despite a meta-regression analysis that found no clear relationship between aspirin dose and the risk of gastrointestinal GI) bleeding [5]. A recent task force publication addressed the use of aspirin for the primary prevention of ischemic stroke (3), but the recommendation for the use of aspirin for primary prevention of coronary heart disease (CHD) is less clear; and, the risk/benefit is perhaps an even more important consideration for primary compared to secondary prevention. The US Preventive Services Task Force estimates that for baseline risks of 1%, 3%, and 5%, 1–4, 4–12, and 6–20 CHD events can be avoided with aspirin primary prophylaxis, but at the risk of 0–2 hemorrhagic strokes and 2–4 major GI bleeds [3]. As primary prophylaxis, it is thus not clear at what risk levels the benefits of aspirin use outweigh its risks.

Ethnic and racial differences of aspirin for primary (and secondary) prevention are even less clear than aspirins use overall. For example, acute CHD mortality rates are twice as high in African Americans compared to whites with a larger disparity at younger ages, but little is known about the explanation for these differences. Since there are few reports on aspirin use for primary prevention of acute CHD, and even fewer by race, we examined these relationships in the REGARDS study, a large biracial national cohort.

## 2. MATERIALS AND METHODS

### 2.1 Study Population

REGARDS is a national, population-based, biracial, longitudinal cohort study designed to examine underlying causes for racial and regional differences in stroke and CHD. The study oversampled African Americans (AAs) and persons living in the Stroke Belt region of the United States. Between January 2003 and October 2007, 30,239 individuals were enrolled, including 42% AA, 58% white, 45% men and 55% women. The sample includes 21% of participants from the Stroke Belt/Buckle, 35% from the Stroke Belt states, and the remaining 44% from the other 40 contiguous states. Participants were selected from commercially available lists (Genesys) based upon the above regions, and age of 45 years and over. A brochure informed participants of the study and an upcoming phone call. During that call, verbal consent was obtained and a questionnaire was administered. The telephone response rate was 33%; the cooperation rate among those with confirmed eligibility was 49% (similar to the Multi-Ethnic Study of Atherosclerosis).

A participant was considered enrolled if they completed the telephone questionnaire and the in-person physical examination. Computer-assisted telephone interview (CATI) methods to collect demographic information and medical history were obtained by trained interviewers. Consent was obtained verbally by telephone and subsequently in writing during a follow-up in-home visit. The physical exam included anthropometric and blood pressure measurements, blood samples, and an electrocardiogram was conducted in-person, 3–4 weeks after the telephone interview. A medication inventory was conducted via pill bottle review at the time of the in-home visit. Self-administered questionnaires were left with the participant to gather further information. Participants were followed by telephone at six-month intervals and a report of a potential event triggered medical record retrieval; and reports of death triggered interviews with proxies in addition to retrieval of any hospital records that corresponded to a hospitalization near the time of death. The National Death Index was also queried. Study methods were reviewed and approved by all involved Institutional Review Boards. Additional methodological details are provided elsewhere [6]. For this analysis, the 5314 individuals with self-reported CHD at baseline (MI or coronary intervention) or evidence of MI on the baseline study ECG were excluded, since the focus was on primary prevention.

The primary dependent variable was incident acute CHD, defined as nonfatal or fatal events as adjudicated by a central panel of experts. Definitions of the outcomes were based on international consensus [7]. MI was classified as definite or probable if there was a biomarker (almost always troponin) rising or falling pattern with the peak ≥ twice the lowest listed upper limit of normal, plus at least one of the following: symptoms or signs suggestive of ischemia or EKG changes consistent with acute ischemia. If there were diagnostic EKG changes and ischemic signs or symptoms present but biomarkers were either unavailable or equivocal, the event was classified as probable MI. Acute CHD death was defined as definite fatal MI if death was within 28 days of hospital admission in definite MI cases, or postmortem findings consistent with MI within 28 days of death; while probable fatal MI was defined as death within 28 days of hospital admission in cases defined as probable MI; or death within 6 hours of hospital admission with cardiac symptoms and/or signs when other confirmatory data (biomarkers, ECG) were absent or not diagnostic. The designation of probable CHD death was applied to out-of-hospital cases of sudden cardiac death, or a death suggestive of acute CHD with a history of CHD and no other plausible cause of death. Outcomes that were analyzed included incident acute CHD, which included both fatal and nonfatal events, and fatal or nonfatal incident CHD were analyzed separately.

The primary independent variable was aspirin use. A participant was considered a “regular aspirin user” if they answered affirmatively to the question “Are you currently taking aspirin or aspirin containing products regularly, that is, at least two times each week?” Among these regular aspirin users, those answering affirmatively to: “For what purpose are you taking aspirin? Is it to reduce the chance of a heart attack or stroke?” were considered prophylactic aspirin users for the primary analysis. Those answering affirmatively to the following question: “Is it to relieve pain?” And these participants were analyzed as aspirin users in a sensitivity analysis.

Factors considered as potentially confounding the relation between race and aspirin use were grouped into demographic measures, measures of socio-economic status, and cardiovascular risk factors. Demographic factors included age (defined in 10-year strata starting with age 45), race and gender. Measures of socio-economic status included annual household income and education (defined in strata, see Table 1). Cardiovascular risk factors included self-reported perceived health (on a 5-point scale from Poor to Excellent), hypertension (SBP≥140mmHg, or DBP ≥90mmHg, or self-reported use of antihypertensive medications), diabetes (fasting glucose ≥126mg/dL or non-fasting glucose ≥200mg/dL or self-reported use of diabetes medications), dyslipidemia (total cholesterol ≥240mg/dL, low-density lipoprotein cholesterol ≥160mg/dL, high density lipoprotein cholesterol ≤40mg/dL or self-reported use of lipid lowering medications), smoking status (never, past, or current), and alcohol use (never, past, or current). Measures of medication adherence, were assessed by the validated Morisky scale* [8].

We addressed confounding that could stem from individuals at highest risk possibly being the most motivated to take prophylactic aspirin. This potential confounding could result in subjects being categorized at high risk in analyses, so we conducted an analysis that examined risk by quartiles of the Framingham Coronary Heart Disease Risk Score (FRS) [9,10] which was used as a summary index of the CHD risk factor burden for each participant. This score reflects the 10-year probability of CHD given the individual's demographic and risk factor profile. The FRS includes age, sex, systolic blood pressure, diastolic blood pressure, total cholesterol, high-density cholesterol, diabetes and current cigarette smoking.

### 2.2 Statistical Analysis

The primary goal was to assess differences in risk for incident acute CHD by prophylactic aspirin usage. Univariate differences were tested using the chi-square test. To estimate the risk associated of treatment with prophylactic aspirin, we constructed Cox proportional hazards models to derive hazard ratios and 95% confidence intervals. Incremental models were used to test the impact that potential confounding variables had on any observed associations. We first adjusted for demographic factors (age, race, sex, and region), then adding indices of SES (income and education), then perceived general health, self-reported CVD risk factors (hypertension, diabetes, dyslipidemia, cigarette smoking and alcohol use), and finally the FRS.

To further assess the potential for a differential effect within patient subgroups (i.e., effect modification), we did a series of additional analyses, including an analysis stratified by FRS, race, and gender. We also analyzed risks associated with time to incident nonfatal and fatal acute CHD assessed separately, and an analysis where regular aspirin use for pain was included (Regular Aspirin Use-Table 1). Last, we examined time to all-cause mortality as a summary measure balancing risks and benefits. Statistical calculations were carried out with SAS version 9.2 (Cary, NC).

## 3. RESULTS

**Fig. 1:**
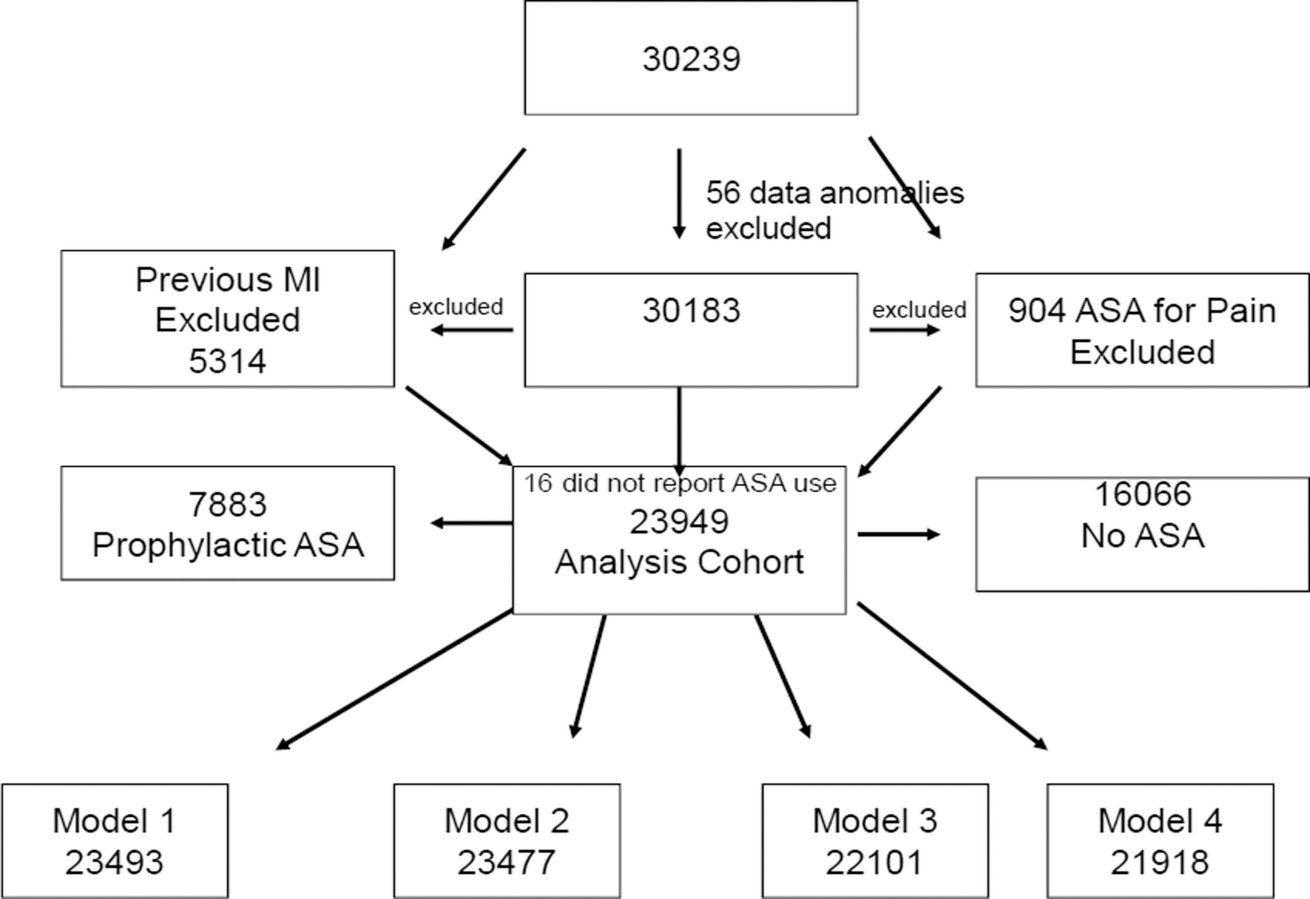
Exclusionary MI cascade

Of the total REGARDS sample, 5314 had a history or evidence of CHD at baseline and were excluded from the primary analysis. Sixteen individuals who did not answer the questions about aspirin use were excluded, and the 904 reported regularly taking aspirin for pain were excluded from the primary analysis. This left an analytic sample of 23,949. In multivariable analysis, other variables were missing in small numbers for each model {(model 1 consisted of 23493; model 2 of 23477, model 3 of 22101 and model 4 of 21918, (Fig. 1)}.

The characteristics of the study sample are shown in (Table 1). As can be seen, the number of users of aspirin for prophylaxis in the sample was 7883, or 33% of the total analyzable population. Also noted in this Table is that there were 503 acute incident CHD events.

The results of the primary multivariable analysis are presented in (Table 2). In all models, over a mean follow-up of 3.5 years (maximum 6 years) prophylactic aspirin use was not associated with incident acute CHD relative to no use. For the first model including only demographics, the hazard ratio (HR) was 1.05 (95% CI 0.86, 1.29). Adding socioeconomic indicators to this model had little impact on the HR. Beyond that, expected HRs for age, race, gender, income, level of education, diabetes, perceived health status etc. demonstrated expected trends.

In the analysis stratified by risk score, prophylactic aspirin use did not demonstrate any association with incident acute CHD (Table III). Of those using aspirin for prophylaxis, the majority (75%) was utilizing a low dose of 180 mg/d or less and, the use of low dose aspirin was slightly more common in whites (77.7%) than in African Americans (71.4%), and in women (78.8%) than men (71.4%). Use of low dose prophylactic aspirin was also more common among those of a higher socio-economic status (higher income and more education), those without diabetes or hypertension, never smokers, and current alcohol drinkers or those never having alcohol intake. Aspirin dose was similar among those with and without dyslipidemia (p>0.05). Subjects taking higher aspirin doses had higher Framingham Stroke and Coronary Heart Disease Risk Scores.

Sensitivity analysis that included the 904 subjects who reported taking regular aspirin for pain (rather than for prophylaxis), did not appreciably change the results (data not shown). However, in analyses stratified by FRS quartile, one unexpected finding was a higher incidence of acute CHD in low risk aspirin users. This latter unexpected finding, as well as the overall findings, held when we included all aspirin use, all-cause mortality, measures of medication adherence, and SBP. The findings did not differ by gender and diabetes status (tests of interaction were not significant). These findings were similar for fatal and nonfatal CHD analyzed separately, and for all-cause mortality (data not shown). Recent guidelines have suggested certain subsets that might benefit from prophylactic aspirin use such as women >65yo and women who are either high risk or diabetic. In these aforementioned subsets, aspirin did not affect the acute CHD endpoints.

The potential for aspirin to be associated with cerebral and gastrointestinal (GI) bleeding was also explored. On the CATI the question was asked "For each time you/he/she stayed as a patient in a hospital, nursing home or rehabilitation center, I need to know the reason, the name and location of where you/he/she stayed, the date and the name of the doctor who treated you/him/her. Since we last spoke to you/him/her?

What was the reason you/he/she were/was admitted?

1. Stroke/brain aneurysm/TIA
2. Heart-related condition
3. Cancer
4. Other specify

If answered other, we searched the free text "other specify" response for the word "bleed" and then two of the authors classified each of the bleeds as a GI or not. For cerebral bleeds, the hospital records were centrally adjudicated by an expert committee, and no statistically significant difference was observed between aspirin users and non users. For GI bleeds (n=210), two models showed no difference but one model was borderline significant (p<.0552).

## 4. DISCUSSION

In this large national cohort of subjects free of baseline CHD, we found no association between prophylactic aspirin use and incident acute CHD over a mean follow-up of 3.5 years. This lack of association was seen regardless of age, race, sex, degree of CHD risk factor burden, and a number of other demographic and baseline disease variables. Additional analyses examining risks associated with fatal incident events, and with all-cause mortality, revealed a similar lack of association. No excess hospitalized bleeding was observed in aspirin (mostly using low doses) vs. non aspirin users.

Since cardiovascular disease is the leading cause of death, efforts have been ongoing to identify interventions that contribute to CVD prevention [11]. The role of prophylactic aspirin use for the primary prevention of CHD is one such effort, but its use has not been convincingly established, although aspirin is approved for primary prevention in over 35 countries [12].

A recent meta-analysis concluded that primary prevention with aspirin decreased the risk for total cardiovascular events and nonfatal MI, but there were no significant differences in the incidence of stroke, CVD mortality, all-cause mortality or total CHD [13]. The 3 most recent studies in that meta-analysis were, for the most part inconclusive. Because of continued controversy regarding the exact role of prophylactic aspirin, there is an ongoing large clinical trial being conducted in a population at moderate risk of initial events, the Aspirin to Reduce Risk of Initial Vascular Events (ARRIVE) [4]. This latter study includes approximately 12,000 patients in seven countries and over 400 study sites. ARRIVE’s aims are to demonstrate the efficacy and safety of low-dose (100-mg daily) aspirin in preventing first events associated with CVD in moderate risk individuals (defined as a 10–20% 10-year risk of a CHD event). Since many past trials assessed the role of aspirin in low risk subjects, and since we wanted to examine the role of confounding by baseline risk, we did stratify the analysis by baseline risk and found no association at any CHD risk level with one exception–the finding that a higher incidence of acute CHD was observed in low risk aspirin users. Given the multiple analyses we conducted, it is possible that this exception is a chance result but it may be worthwhile to examine this in other cohorts.

Our study has several limitations worth noting. Some non-laboratory risk factors were based on self-report (although this is common to many epidemiologic studies), and individuals without telephones were necessarily excluded from selection into the study population. These excluded individuals may be of lower socioeconomic status and, therefore, may have different risk factor profiles than those included in this analysis. Also, the mean follow-up time of 3.5 years may not be long enough to have shown any benefit. Finally, participant usage of aspirin was also by self-report as was medication adherence.

## 5. CONCLUSION

In conclusion, in this large national cohort study, we observed no association over a mean follow-up time of 3.5 years, between self-reported use of aspirin as primary prophylaxis in the prevention of incident acute CHD overall, nor on incident nonfatal or incident fatal acute CHD, or all-cause mortality. We also did not observe any excess hospitalized bleeding (cerebral or GI).

## Figures and Tables

**Table 1:** Data broken down by prophylactic vs no aspirin use

Table 1	Overall		Not prophylactic aspirin user		Prophylactic aspirin user		p-value of prophylactic aspirin users vs. no	Not regular aspirin user		Regular aspirin user		p-value of regular aspirin users vs. no
	
	N	%	N	%	N	%		N	%	N	%	
	
Overall	23949		16066	67.1	7883	32.9		15506	64.8	8443	35.3	
**Acute CHD**												
No	23446	97.9	15757	67.2	7689	32.8	0.0064	15213	64.9	8233	35.1	0.0021
Yes	503	2.1	309	61.4	194	38.6		293	58.3	210	41.8	
**Mortality risk region**												
Low	8083	33.8	5340	66.1	2743	33.9	0.0044	5160	63.8	2923	36.2	0.0073
Medium	8311	34.7	5547	66.7	2764	33.3		5346	64.3	2965	35.7	
High	7525	31.5	5153	68.5	2372	31.5		4976	66.1	2549	33.9	
**Race**												
Black	10281	42.9	7259	70.6	3022	29.4	<.0001	7009	68.2	3272	31.8	<.0001
White	13668	57.1	8807	64.4	4861	35.6		8497	62.2	5171	37.8	
Gender												
Male	9986	41.7	6223	62.3	3763	37.7	<.0001	5951	59.6	4035	40.4	<.0001
Female	13963	58.3	9843	70.5	4120	29.5		9555	68.4	4408	31.6	
**Age group**												
45–55	3359	14.0	2751	81.9	608	18.1	<.0001	2714	80.8	645	19.2	<.0001
55–65	9598	40.1	6609	68.9	2989	31.1		6444	67.1	3154	32.9	
65–75	7375	30.8	4518	61.3	2857	38.7		4300	58.3	3075	41.7	
75–85	3223	13.5	1953	60.6	1270	39.4		1829	56.8	1394	43.3	
85+	394	1.6	235	59.6	159	40.4		219	55.6	175	44.4	
**Income**												
<$20K	4089	17.1	2811	68.8	1278	31.3	0.0002	2684	65.7	1405	34.4	0.0239
$20K–$35	5664	23.7	3807	67.2	1857	32.8		3676	64.9	1988	35.1	
$35K–$75K	7205	30.1	4857	67.4	2348	32.6		4703	65.3	2502	34.7	
$75K+	3992	16.7	2559	64.1	1433	35.9		2495	62.5	1497	37.5	
Missing income	2999	12.5	2032	67.8	967	32.2		1948	65.0	1051	35.1	
**Years of education**												
< High School	2780	11.6	1882	67.7	898	32.3	<.0001	1789	64.4	991	35.7	<.0001
High School	6076	25.4	4179	68.8	1897	31.2		4017	66.1	2059	33.9	
Some College	6483	27.1	4458	68.8	2025	31.2		4318	66.6	2165	33.4	
College+	8593	35.9	5534	64.4	3059	35.6		5370	62.5	3223	37.5	
**Perceived health**												
Excellent	4246	17.8	2929	69.0	1317	31.0	0.0005	2831	66.7	1415	33.3	0.0006
Very Good	7663	32.1	5160	67.3	2503	32.7		4982	65.0	2681	35.0	
Good	8248	34.5	5520	66.9	2728	33.1		5329	64.6	2919	35.4	
Fair	3118	13.0	2037	65.3	1081	34.7		1959	62.8	1159	37.2	
Poor	626	2.6	386	61.7	240	38.3		373	59.6	253	40.4	
**Hypertenision**												
No	10435	43.7	7752	74.3	2683	25.7	<.0001	7560	72.5	2875	27.6	<.0001
Yes	13461	56.3	8281	61.5	5180	38.5		7914	58.8	5547	41.2	
**Diabetes**												
No	18507	80.4	12777	69.0	5730	31.0	<.0001	12375	66.9	6132	33.1	<.0001
Yes	4519	19.6	2610	57.8	1909	42.2		2472	54.7	2047	45.3	
**Dyslipidemia**												
No	10245	44.6	7483	73.0	2762	27.0	<.0001	7219	70.5	3026	29.5	<.0001
Yes	12750	55.4	7876	61.8	4874	38.2		7597	59.6	5153	40.4	
**Smoke status**												
Never	11322	47.5	7734	68.3	3588	31.7	<.0001	7486	66.1	3836	33.9	<.0001
Past	9126	38.3	5810	63.7	3316	36.3		5572	61.1	3554	38.9	
Current	3408	14.3	2454	72.0	954	28.0		2382	69.9	1026	30.1	
**Alcohol use**												
Never	7354	30.7	5046	68.6	2308	31.4	0.0005	4835	65.8	2519	34.3	0.0425
Past	4034	16.8	2731	67.7	1303	32.3		2628	65.2	1406	34.9	
Current	12561	52.4	8289	66.0	4272	34.0		8043	64.0	4518	36.0	
**Framingham cardiac risk score**												
0–5	8500	37.7	6183	72.7	2317	27.3	<.0001	6032	71.0	2468	29.0	<.0001
5–10	6212	27.5	4028	64.8	2184	35.2		3874	62.4	2338	37.6	
10–20	5155	22.9	3203	62.1	1952	37.9		3081	59.8	2074	40.2	
20+	2690	11.9	1689	62.8	1001	37.2		1588	59.03	1102	41.0	

**Table 2:** Incremental analyses

Incident acute nonfatal (definite/probable) OR fatal CHD (definite/probable, in- and out-of- hospital), on/before 12/31/2008	Demographics			Demo+SES			Demo+SES+CD+RF			Demo+SES+CD+RF +FH		

	HR	LL	UL	HR	LL	UL	HR	LL	UL	HR	LL	UL
Prophylactic aspirin vs. no	1.054	0.864	1.287	1.09	0.893	1.331	0.998	0.81	1.23	1.073	0.872	1.321
MI medium region vs. low	1.17	0.927	1.478	1.139	0.901	1.439	1.145	0.897	1.462	1.147	0.897	1.465
MI high region vs. low	1.118	0.876	1.426	1.089	0.853	1.389	1.098	0.852	1.414	1.081	0.838	1.394
Black vs. white	1.175	0.965	1.431	0.975	0.793	1.198	0.785	0.629	0.981	0.858	0.689	1.068
Male vs. female	2.264	1.858	2.76	2.537	2.07	3.108	2.651	2.119	3.316	1.714	1.339	2.193
55–65 vs.45–55	1.9	1.241	2.909	1.864	1.217	2.855	2.095	1.305	3.363	1.664	1.03	2.687
65–75 vs.45–55	2.536	1.656	3.884	2.278	1.482	3.504	2.712	1.678	4.384	1.813	1.105	2.977
75–85 vs.45–55	3.845	2.468	5.992	3.221	2.051	5.06	3.954	2.392	6.537	2.248	1.326	3.81
85+vs.45–55	4.251	2.131	8.477	3.518	1.751	7.069	4.4	2.027	9.554	2.122	0.955	4.716
$20K–$35K vs.< $20K	.	.	.	0.807	0.612	1.064	0.858	0.642	1.145	0.87	0.651	1.162
$35K–$75K vs< $20K	.	.	.	0.578	0.425	0.786	0.658	0.477	0.907	0.676	0.49	0.933
$75K+ vs.< $20K	.	.	.	0.607	0.413	0.892	0.75	0.498	1.129	0.782	0.518	1.181
Missing income vs.< $20K	.	.	.	0.666	0.467	0.948	0.69	0.473	1.008	0.705	0.483	1.03
High school graduate vs. less than high school graduate	.	.	.	0.663	0.489	0.899	0.743	0.541	1.02	0.772	0.562	1.062
some college vs. less than high school	.	.	.	0.51	0.366	0.712	0.667	0.47	0.947	0.689	0.485	0.98
college graduate vs. less than high school	.	.	.	0.914	0.679	1.231	0.998	0.73	1.364	1.048	0.765	1.436
Very good health vs. excellent	.	.	.	.	.	.	0.906	0.646	1.272	0.941	0.671	1.32
Good health vs. excellent	.	.	.	.	.	.	1.202	0.866	1.669	1.288	0.931	1.78
Fair health vs. excellent	.	.	.	.	.	.	1.576	1.081	2.298	1.746	1.206	2.526
Poor health vs. excellent	.	.	.	.	.	.	1.988	1.146	3.449	2.186	1.269	3.765
Hypertensive vs. not	.	.	.	.	.	.	1.42	1.128	1.788	0.93	0.75	1.154
Diabetic vs. not	.	.	.	.	.	.	1.521	1.208	1.915			
Dyslipidemic vs. not	.	.	.	.	.	.	1.043	0.843	1.29	1.092	0.86	1.388
Past Smoker vs. never	.	.	.	.	.	.	1.104	0.869	1.401	1.097	0.802	1.501
Current smoker vs. never	.	.	.	.	.	.	1.704	1.267	2.291	0.991	0.764	1.287
Current alcohol vs. never	.	.	.	.	.	.	0.957	0.737	1.242	1.244	0.927	1.67
Past alcohol vs. never	.	.	.	.	.	.	1.198	0.894	1.607	2.221	1.522	3.242
CHD risk quart 2 vs. 1							.	.	.	3.162	2.145	4.661
CHD risk quart 3 vs. 1							.	.	.	4.915	3.204	7.54
CHD risk quart 4 vs. 1							.	.	.	5.193	2.995	9.004

**Table 3:** Time to incident acute nonfatal (definite/probable) or fatal CHD (definite/probable*), stratified by risk score

Time to incident acute nonfatal (definite/probable) OR fatal CHD (definite/probable, in- and out-of-hospital), on/before 12/31/2008 stratified by risk score	Model 1 prophylactic aspirin, region, race, gender, age			Model 2 model 1+income, education			Model 3 model 2+dyslipidemic, hypertensive, diabetic, self reported health, drinking status, smoking status		

	****All hazard ratios and limits are prophylatic aspirin users vs. not								

	Hazard Ratio	Lower Limit	Upper Limit	Hazard Ratio	Lower Limit	Upper Limit	Hazard Ratio	Lower Limit	Upper Limit
CHD risk score 20+	1.189	0.825	1.714	1.218	0.845	1.756	1.187	0.823	1.713
CHD risk score 10–20	0.714	0.49	1.04	0.739	0.507	1.078	0.691	0.471	1.014
CHD risk score 5–10	1.126	0.741	1.712	1.136	0.748	1.727	1.078	0.708	1.642
CHD risk score 0–5	2.008	1.083	3.725	2.002	1.079	3.713	1.963	1.049	3.672

* in- and out-of-hospital
